# Job vacancies at the European Centre for Disease Prevention and Control (ECDC)

**DOI:** 10.2807/1560-7917.ES.2022.27.7.2202171

**Published:** 2022-02-17

**Authors:** Eurosurveillance editorial team

**Affiliations:** 1 European Centre for Disease Prevention and Control (ECDC), Stockholm, Sweden

The European Centre for Disease Prevention and Control (ECDC) invites applications for the following positions:

 

**Principal Expert Sexually Transmitted Infections, HIV or Tuberculosis / Group Leader Sustainable Development Goal-Targeted Diseases (DPR)**
The application deadline expires on 11 Mar 2022.

 

**Principal Expert Emergency Preparedness and Response / Group Leader Emergency Preparedness and Response**
The application deadline expires on 14 Mar 2022.

 

For more information, please visit the ECDC website: 

https://ecdc.europa.eu/en/work-us/vacancies?f%5B0%5D=deadline_date%3A1

